# Clinical Studies Applying Cytokine-Induced Killer Cells for the Treatment of Gastrointestinal Tumors

**DOI:** 10.1155/2014/897214

**Published:** 2014-01-16

**Authors:** Clara E. Jäkel, Annabelle Vogt, Maria A. Gonzalez-Carmona, Ingo G. H. Schmidt-Wolf

**Affiliations:** ^1^Center for Integrated Oncology (CIO), University Medical Center Bonn, Sigmund-Freud-Straße 25, 53127 Bonn, Germany; ^2^Department of Internal Medicine I, University Medical Center Bonn, Sigmund-Freud-Straße 25, 53127 Bonn, Germany

## Abstract

Tumors of the gastrointestinal system represent a significant share of solid tumors worldwide. Despite the advances in diagnosis and treatment, the prognosis of gastrointestinal tumors is still very poor and improved therapies are indispensable. Cytokine-induced killer (CIK) cells are feasible for an immunotherapeutic approach as they are easily available and have an advantageous biologic profile; they are rapidly proliferating and their high cytotoxicity is non-MHC-restricted. We summarize and discuss twenty recent clinical studies applying CIK cells for the treatment of gastric, pancreatic, hepatocellular, and colorectal cancer. Autologous CIK cells were transfused intravenously, intraperitoneally, or via the common hepatic artery. In all studies side effects and toxicity of CIK cell therapy were mild and easily controllable. The combination of CIK cell therapy with conventional adjuvant or palliative therapies was superior to the standard therapy alone, indicating the benefit of CIK cell therapy for cancer patients. Thus, CIK cells represent a promising immunotherapy for the treatment of gastrointestinal tumors. The optimal treatment schedule and ideal combination with conventional therapies should be evaluated in further clinical studies.

## 1. Introduction

Tumors of the gastrointestinal (GI) system constitute a major part of the cancer incidence and mortality statistics. Worldwide, colorectal cancer is the most frequent type of GI cancer: it is the third most common cancer in men and the second most common in women. Moreover, colorectal cancer accounts for the largest share of GI cancer-related deaths in women, while liver cancer is the most common cause of death among GI tumors of men [1].

Despite the recent advances in diagnosis and therapy, outcomes for patients with GI tumors remain very poor. Often, GI tumors are diagnosed only at advanced stages due to the lack of specific symptoms and screening methods. As a result, 5-year survival rates are low [2–5].

Adoptive cell immunotherapy might be used in combination with standard therapies—as adjuvant postsurgical treatment and as palliative treatment—to improve survival and quality of life of GI cancer patients. Cytokine-induced killer (CIK) cells have the best credentials to be effective in this therapeutic approach. Compared to lymphokine-activated killer (LAK) cells, CIK cells can be obtained more easily and reveal a higher tumor-specific cytotoxic activity [6–10]. Similarly, there are several factors hampering the adoptive cell therapy with tumor-infiltrating lymphocytes (TILs), for example, the difficulty to recover sufficient quantities of these cells and their poor migration to the tumor side [11, 12].

CIK cells can be easily developed from peripheral blood lymphocytes (PBLs) and stimulated with interferon-*γ* (IFN-*γ*), monoclonal antibody against CD3 and interleukin (IL)-2. The heterogeneous cell population gains its potent, nonmajor histocompatibility complex (MHC)-restricted cytotoxicity mainly through expansion of CD3^+^CD8^+^CD56^−^ cells to CD56-positive natural killer (NK) T cells [10, 13, 14]. CIK cell cytotoxicity is mediated by perforin release and dependant on NKG2D recognition and signaling [15, 16]. The addition of high doses of IL-2 during generation of CIK cells is critical for the expression of the NKG2D adapter protein DAP10 which in turn is essential for cytolysis [16]. NKG2D ligands (e.g., MICA, MICB, and ULBP 1–4) are expressed on both solid and hematologic tumors [17–19].

In the following sections, clinical studies applying CIK cells for the treatment of GI tumors are reviewed and discussed.

## 2. Gastric Cancer

Gastric cancer is the third leading cancer-related cause of death among men and the fifth most common among women. It is therefore a major health issue worldwide. Apart from dietary aspects, *Helicobacter pylori*, a bacterium colonizing the stomach, is the most prominent known risk factor for gastric cancer [1, 20]. Today, combinations of surgical resection, different platin, fluoropyrimidine, and taxane-derived chemotherapies and radiotherapy are the standard treatment options for patients with stomach cancer. At the time of diagnosis these patients generally present with advanced or even metastatic disease. Thus, the cure rates remain poor and novel treatment strategies are required [1].

**Figure 1:**
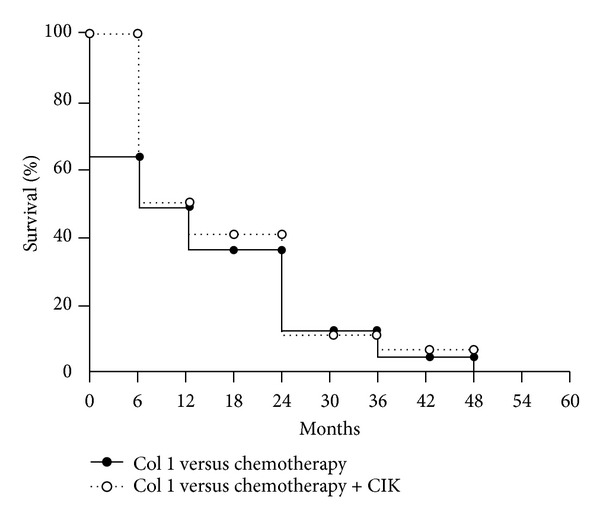
Cumulative survival rate of the patients analyzed by the Kaplan-Meier method. No patient was alive after five years (modified from [21]).

Jiang et al. provide a study applying CIK cells in combination with chemotherapy in patients with advanced gastric cancer who had all undergone palliative gastrectomy [21]. After gastrectomy, twenty-five patients were treated with three cycles of folinic acid, 5-fluorouracil (5-FU), and oxaliplatin (FOLFOX) chemotherapy. Thirty-two patients were treated with FOLFOX chemotherapy plus autologous CIK cell therapy consisting of five transfusions of CIK cells (1 × 10^9^) after each chemotherapy. In both treatment groups, serum levels of tumor markers were significantly (*P* < 0.05) lower after therapy. The decrease was more pronounced in the patient group receiving additional CIK cell therapy. In the beginning, there was an increase in the cumulative survival rate of the patients treated with CIK cell transfer but after two years, there was no difference in survival between the two groups (Figure 1). Still, the authors conclude that there is a benefit of combined chemo- and CIK cell therapy for patients with advanced gastric cancer.

A similar study was conducted by Shi et al. a few years later [22]. The final analysis included 151 patients with gastric cancer in locally advanced stage. All patients had undergone gastrectomy. During the generation of CIK cells *in vitro*, the number of CD3^+^CD56^+^ cells increased severely (on average 700-fold) and all cell populations used for immunotherapy had no less than 30% CD3^+^CD56^+^ cells. Patients received six cycles of multidrug adjuvant chemotherapy based on 5-FU. Seventy-four patients additionally received at least three cycles of autologous CIK cell therapy (immunotherapy group). One cycle consisted of five CIK transfusions.

One week after immunotherapy the mean percentages of CD3^+^ and CD4^+^ cells and the CD4^+^/CD8^+^ ratio increased in the patients' blood and remained at a higher level for up to two months (after three cycles of CIK cell therapy). All side effects occurring during or after transfusion could easily be treated with symptomatic therapy in case they did not resolve on their own within 24 hours. The most common side-effects were fever, chills, headache, rash, nausea, and vomiting (ranging from 5.0 to 20.8%).

The median followup was 50.5 months; in the end 137 patients (90.7%) had died and 143 (94.7%) had relapsed (mostly hematogenous recurrence). The disease-free survival (DFS) rate was significantly better (*P* = 0.044) in the immunotherapy group than in the control group. A trend towards an improved overall survival (OS; *P* = 0.071) could be observed in the immunotherapy group as well. Moreover, by retrospective subgroup analysis, patients with intestinal-type tumors could be found to benefit most from CIK cell therapy (OS: *P* = 0.045; DFS: *P* = 0.023; diffuse or mixed-type tumors: OS: *P* = 0.970; DFS: *P* = 0.962).

On the whole, CIK cell immunotherapy prolonged the DFS in patients with locally advanced stomach cancer and also the OS in patients with intestinal-type tumors. Therefore, the intestinal-type tumor might be a prospective inclusion criterion for CIK cell immunotherapy.

Wang et al. published a study combining capecitabine and oxaliplatin chemotherapy with intraperitoneal (i.p.) perfusion of CIK cells [23]. Forty-two advanced gastric cancer patients with ascites were enrolled in two groups: the chemotherapy group (22 patients) and the combination group (chemotherapy plus CIK perfusion; 20 patients).

The combination of chemotherapy and CIK cells was well tolerated, and there were no serious adverse reactions after CIK perfusions. Compared to chemotherapy alone, the combined therapy was able to reduce the volume of 2-cycle peritoneal fluid drainage (*P* = 0.018). Patients additionally treated with CIK cells showed a longer median time to progression (TTP; *P* = 0.001) and a superior OS (*P* = 0.006).

Another study that was published in 2008 focuses on the side effects occurring during CIK cell treatment [24]. Sixty elderly patients with advanced gastric cancer were treated with FOLFOX chemotherapy; 29 of them received additional intravenous (i.v.) autologous CIK cell infusions.

Side effects appearing after CIK transfusions included chills (13 patients), fever (9 patients), a general malaise (3 patients), nausea, and vomiting (1 patient). These symptoms could all be managed by symptomatic therapy. The therapeutic results were also quite promising. The total remission rate in the CIK cell therapy patient group was higher than in the group of patients treated with chemotherapy alone (58.6% versus 45.2%). In the CIK cell-treated group, eight patients developed partial remission (PR), nine moderate remission (MR), seven stable disease (SD), and five progressive disease (PD). In the chemotherapy group, only five patients developed PR, nine MR, seven SD, and even ten PD.

The same research group performed a retrospective study to analyze the correlation between CIK cell therapy and cancer-related death in patients with gastric cancer [25]. One hundred and fifty-six patients were included in this study; 81 patients were treated with FOLFOX chemotherapy alone and 75 with additional CIK cell immunotherapy five times after six cycles of chemotherapy.

The 2-year survival time was significantly longer after additional CIK cell therapy than after chemotherapy alone (*P* = 0.007). The 5-year survival rates showed a clear trend towards a superior survival time for patients treated with CIK cells (*P* = 0.0526). The frequency of CIK cell immunotherapy was found to be significantly associated with the survival of patients (*P* = 0.002).

Another retrospective study evaluates the clinical outcome of 165 advanced gastric cancer patients treated with either FOLFOX chemotherapy or 5-FU/cisplatin chemotherapy; patients in the study group were additionally treated with CIK cell therapy [26]. All patients underwent surgery and received four cycles of adjuvant chemotherapy within one month after surgery. While 112 patients in the control group received no additional treatment, 53 patients in the study group were given CIK cell therapy after four cycles of chemotherapy in a one-month interval. CIK therapy was maintained as long as the patients agreed or until tumor progression occurred. Two to twenty CIK therapy cycles were given with a median of three cycles per patient.

No serious side effects were observed after CIK cell transfer. The 3-year progression-free survival (PFS) and OS rates were higher in the CIK cell therapy group compared to the study group, but the differences were not significant. A significant improvement could be seen for the 5-year PFS (49.1% in the study group versus 24.1% in the control group, *P* = 0.026) and for the 5-year OS (56.6% versus 26.8%, *P* = 0.014). The median PFS was 36.0 months in the CIK treated study group versus 23.0 months in the control group (*P* = 0.028). The OS time was also significantly longer in the study group: 96.0 months versus 32.0 months in the control group (*P* = 0.003). Within the study group, the frequency of CIK cell therapy, the clinical stage, and the followup therapy were found to be the most important factors for PFS and OS (*P* < 0.05).

Again, CIK cell therapy was shown to prevent recurrence and improve survival in combination with surgery and chemotherapy. The authors themselves criticize the retrospective character and the number of patients of this study and wish for a prospective paired study to confirm these results. Nevertheless, this study gives a good insight into the possibilities of CIK cell transfer in the adjuvant therapy of gastric cancer.

These six clinical studies show that the combination of CIK cell therapy with palliative or adjuvant chemotherapy protocols can be a significant step forward in the treatment of advanced gastric cancer. The adoptive transfer of CIK cells was well tolerated,—i.v. as well as i.p.—and prolonged survival in all studies discussed above.

The clinical studies on CIK cells for the treatment of gastric cancer are summarized in Table 1.

## 3. Pancreatic Cancer

The incidence of pancreatic cancer is relatively low compared to the other types of cancer discussed here. Nevertheless, it belongs to the ten most common causes of death from cancer [1]. Pancreatic adenocarcinoma derived from glandular tissue of the pancreas is the most common type of pancreatic cancer. Among others, alcohol consumption, smoking, and diet are known to be risk factors. Diabetes, chronic pancreatitis, and obesity are also associated with a higher risk of pancreatic cancer. Even infections, for example, with *H. pylori,* have been found to be related to pancreatic cancer [27, 28]. In more than 70% of the cases, pancreatic adenocarcinoma is diagnosed at advanced stages. Standard therapy includes surgery, chemotherapy (mostly with gemcitabine), and radiotherapy, but much effort is made to develop new molecular therapies [29, 30].

Qiu et al. report on a new approach of pancreatic carcinoma-specific immunotherapy using synthesized *α*1,3-galactosyl epitope-pulsed dendritic cells (DCs) along with CIK cells [31]. In this pilot study, fourteen patients with advanced pancreatic cancer were enrolled and treated with gemcitabine combined with oxaliplatin and radiotherapy.

The aim of this clinical approach was to improve tumor-associated antigen (TAA)-pulsed DC therapy. In humans, there is no *α*-galactosyl (*α*-Gal) epitope on the cell membrane, whereas the natural anti-Gal antibody is present in human serum [32]. Hence, the idea behind this study was that the expression of the *α*-Gal epitope on tumor cells can result in *in situ* binding of natural antibodies. This in turn enhances DC phagocytosis and thereby presentation of TAA to T cells, finally generating an antitumoral immune response. Cancer patients often have low immunity; therefore, CIK cells were used for coculturing with the *α*-Gal-expressing tumor cell lysate-pulsed DCs.

In a first step, metastatic tumor nodules or lymph nodes were surgically obtained from the patients. Using neuraminidase and recombinant *α*1,3-galactosyltransferase, *α*-Gal epitopes were synthesized on freshly isolated tumor cells from these biopsies. The cells were then incubated with natural anti-Gal antibody and finally lysed. DCs, which were induced from peripheral blood mononuclear cells (PBMCs) using granulocyte macrophage colony-stimulating factor (GM-CSF), IL-4, and tumor necrosis factor (TNF)-*α*, were then incubated with the *α*-Gal-expressing tumor cell lysate. CIK cells were prepared from bone marrow samples of the patients, harvested on day twelve, and co-cultured with the lysate-pulsed DCs in presence of IFN-*γ*, anti-CD3, and IL-2 for 72 hours. The DC-CIK cell mixtures were then used for i.v. transfusions.

The first injection was applied one week after completion of the conventional therapies. Then, depending on the availability of tumor samples, one to five further injections were administered once a week with increasing numbers of cells (2 × 10^9^ to 10 × 10^9^ cells per injection).

No serious side effects are reported. Moderate increases of CD3^+^ and CD4^+^ T cells (*P* < 0.05) and significant increases of CD3^+^CD8^+^ T cells, CD3^+^CD45RO^+^ cells, and CD3^+^CD56^+^ cells (*P* < 0.01) could be detected in the patients' peripheral blood one week after the third transfusion, indicating a boosting cellular immunity. These levels returned to the original level (before DC-CIK therapy) six to nine months after the third injection. Moreover, a significant increase in IFN-*γ* secretion by PBMCs (*P* < 0.01) was detected, which remained increased for the followup period of 24 months. Most patients (*n* = 12) showed a positive delayed-type IV hypersensitivity (DTH) reaction after three injections. The carcinoembryonic antigen (CEA) and carbohydrate antigen (CA) 19-9 levels decreased in most patients.

The clinical response was evaluated according to RECIST criteria one month after the third injection. Six patients had SD, two PR, and six remained in PD. Interestingly, there were strong correlations between an increasing DTH reaction, a decrease in CEA and CA19-9 levels, respectively, and survival (*P* < 0.01). The four patients who survived the followup period showed strong DTH reactions and significantly increased IFN-*γ* secretion.

This study gives insight into an innovative strategy on pancreatic cancer therapy. Unfortunately, a control group without CIK therapy is missing, which inhibits definite conclusions. Comparing the clinical efficacy of this DC-CIK approach to CIK-therapy alone is also indispensable.

Beyond this study, only one case report on CIK cell therapy for advanced pancreatic carcinoma is published [33]. An elderly patient was diagnosed with a poorly differentiated adenocarcinoma. One month after resection a nodule emerged at the resection margin. Because the patient was unable to tolerate the side-effects of conventional chemo- and radiotherapy, CIK cell immunotherapy was applied. The patient received four i.v. CIK cell infusions of 5 × 10^9^ cells and two million units of IL-2 per infusion from day one to five.

No adverse reactions were observed and the nodule shrank significantly. The formerly elevated levels of CEA, CA 19-9, and CA-724 also reduced to normal values. Following another twelve CIK cell transfusions, the nodule further decreased and marker levels remained normal. After another sixteen injections, the nodule almost disappeared. Finally, the patient received another four cycles to assure treatment efficacy. Unfortunately, a nodule emerged at the same place only a few months later and CA 19-9 levels were elevated again. Altogether the patient had a relatively long PFS of >19 months and an improved quality of life.

This case report gives a promising view on an individual course of CIK cell therapy in a patient with pancreatic cancer. The patient was at poor health and not able to receive chemo- or radiotherapy. Here, CIK cell therapy proved to be a good if not superior alternative.

## 4. Hepatocellular Carcinoma

Hepatocellular carcinoma (HCC) is the most common histological type of liver cancer and the fifth most common cause of malignancy worldwide. Liver cancer rates are more than twice as high in men as in women and the second most common cause of cancer-related death in men. The majority of cases occur in developing countries, where liver cancer is strongly related to infections with hepatitis B and C viruses (HBV and HCV). In industrial countries, where the incidence of HCC is increasing, the most common risk factors are alcohol abuse and obesity resulting in fatty-liver disease, liver fibrosis, and finally liver cirrhosis. In 80% of the cases, HCC is developing on top of a liver fibrosis/cirrhosis [1].

In most of the cases HCC is diagnosed in an already advanced stage or with decompensated liver cirrhosis. Therefore, the majority of patients can only be treated with palliative therapies. Despite the evolving new medical treatments such as the tyrosine kinase inhibitor sorafenib, therapy of HCC is still challenging.

In 2000 a study investigating postsurgical recurrence rates in HCC patients was published [34]. HCC patients who had all undergone hepatic resection were randomly divided into two groups: one group received adjuvant cytokine-stimulated lymphocyte immunotherapy; the other group received no additional treatment. Peripheral blood was drawn from the patients one day before surgery and lymphocytes were cultured with IL-2 and anti-CD3 for two weeks. Patients in the immunotherapy group received autologous lymphocytes at weeks two, three, four, twelve, and 24 after surgery.

In the end, 72 patients received all five CIK cell transfers, two received only four courses of CIK therapy because of detection of extrahepatic metastases, and two received only one cycle and refused subsequent cell infusions. Adverse events occurred in 45 patients and were all WHO grade 1 or 2 and self-limiting.

The median time for followup was 4.4 years. The recurrence rate of HCC was significantly lower in the immunotherapy group (59%, 45 patients) than in the control group (77%, 57 patients; *P* = 0.01). Also the time to first recurrence was significantly longer in the immunotherapy group (*P* = 0.008). Hence, adjuvant cell therapy was able to lower the frequency of recurrence and to extend the recurrence-free time after hepatic resection.

Four years later a small clinical trial about CIK cell therapy in HCC patients was published by Shi et al. [35]. They enrolled thirteen patients with diagnosed HCC. All patients had liver cirrhosis and more than twenty years of chronic HBV infection.

Autologous CIK cells were reinfused i.v. at days ten, thirteen, and fifteen of CIK cell culture. Before treatment and ten days after CIK cell therapy, the patients' PBMCs were analyzed using a flow cytometer: percentages of CD3^+^CD8^+^, CD25^+^, and particularly of CD3^+^CD56^+^ cells were significantly increased (*P* < 0.05). Patients were followedup for up to 108 days after CIK cell therapy. At that time, the composition of lymphocyte subpopulations was still similar to the levels determined ten days after therapy. This indicates that the induced changes in the subpopulations can last for at least 108 days.

As all patients had a background of chronic HBV infection, the influence of CIK therapy on the HBV viral load was of great interest: on average, the HBV content decreased from 1.85 × 10^6^ to 1.41 × 10^5^ copies of DNA/mL three months after therapy. It is well established that DC function is suppressed in patients with HCC and chronic HBV or HCV infections [36]. Therefore, the frequencies of DC1, which induce Th_1_ cell differentiation and immunity, and DC2, which induce Th_2_ differentiation and immunogenic tolerance, were analyzed during this study [37]. The percentages of both cell types were significantly increased in blood after CIK cell therapy (*P* < 0.01). These results suggest that CIK cells are able to play a major role not only in tumor treatment but also in restriction of viral infections.

In a study by Zhang et al. seventeen patients were treated with autologous CIK cells [38]. Most patients have had postoperative recurrence and were in need of further treatment.

CIK therapy is reported as being safe, effective, and without side effects. Without giving reasons, only the results of one patient are given in detail in this publication. This patient had recurrent HCC with metastases and was treated with i.v. transfusion of 1.3 × 10^10^ CIK cells. Following the transfusion, the patient had decreased nausea, vomiting, and ascites. After three months a tumor specimen of this patient showed large lymphocyte infiltration—much more than before treatment. The cells were stained for T cell, T memory cell, and mono/macrophage markers and all cell types were increased after CIK therapy. This probably indicates the induction of an antitumor immune response. Unfortunately, no clinical parameters or results are stated and therefore no conclusions on the clinical effect of the CIK cell transfer can be drawn from this study.

Zhao et al. included 64 HCC patients in a CIK cell therapy study [39]. All patients had undergone transarterial chemoembolization (TACE) and additional radiofrequency ablation (RFA). No residual tumor or extrahepatic metastases could be detected. Each of the 33 patients in the study group received eight autologous CIK infusions every three to four weeks either via the peripheral vein or the hepatic artery. The 31 patients in the control group were given no additional treatment. After a relatively short followup of one year, 29 patients in the study group and 23 patients in the control group were recurrence-free. As in the study discussed above, the HBV DNA content was determined before and after treatment. In the study group, the number of patients with a viral DNA content lower than 1 × 10^3^ increased from 19 patients before treatment to 29 afterwards. In the control group, the viral DNA content of only one patient dropped below 1 × 10^3^.

Again, this study gives an idea of what adoptive CIK cell therapy is capable of; it may reduce recurrence, prolong recurrence-free time, and fight HBV. However, the relatively short followup makes it difficult to draw any substantial conclusions.

The research group conducted a similar study in 2008 [40]. Eighty-five HCC patients were treated by TACE and RFA and were divided into two groups. The 45 patients in the study group received additional CIK cell therapy via the hepatic artery. CIK transfusions were given fortnightly after sequential TACE/RFA, four times as a course of treatment. Thirty-nine patients received eight infusions and six patients received ten infusions. Patients in the control group (*n* = 40) received no additional treatment.

Following the transfusions, eleven patients developed a light fever and shiver; all adverse effects were grade 1 or 2. The proportions of several T cell subsets in the patients' peripheral blood were measured by flow cytometry two weeks after CIK transfusions. CD4^+^, CD3^+^, CD56^+^, and CD3^+^CD56^+^ T cell populations and the CD4^+^/CD8^+^ ratio were significantly increased (*P* < 0.05). Remarkably, the percentages of these cells were lower in patients who experienced recurrence than in patients who were recurrence-free (concerning all 85 patients; *P* < 0.05). Interestingly, the percentage of CD8^+^ T cells, which was decreased after CIK cell therapy, was increased again in recurrent patients.

During eighteen months of followup all patients survived. Fourteen patients (31.1%) of the immunotherapy group had HCC recurrence compared to 32 patients (80.0%) in the control group (*P* = 0.001). The median recurrence-free survival was significantly longer for patients who received CIK cell transfer (*P* = 0.012). Also the recurrence-free survival rates were significantly higher in the immunotherapy group than in the control group (31 patients = 68.9% versus 8 patients = 20.0%; *P* = 0.01).

When entering the study none of the 85 patients showed active lesions or metastases. This study shows that CIK cell immunotherapy can be an effective adjuvant therapy to improve recurrence and survival prognosis for patients with HCC. Thus, CIK cell therapy might be particularly helpful to eradicate microscopic residual tumor lesions to prevent recurrence and improve survival.

A similar study by Pan et al. focuses on changes in serum alpha-fetoprotein (AFP) levels in patients with HCC after TACE/RFA and CIK therapy [41]. AFP is a well-known tumor-associated marker of HCC. This protein is not only known to play a role in the inhibition of the immune system but also in the promotion of cancer cell growth. The aim of this study was to examine AFP as a potential marker to predict the clinical outcome of the combinational therapy of TACE/RFA and CIK cells.

Six to eight weeks after TACE/RFA therapy the possibility of residual tumor burden was excluded by imaging techniques. Before randomization, two consecutive AFP measurements assured a stable AFP level. Patients were then randomly divided into a control group (*n* = 39) and a study group (*n* = 42); patients within the study group received autologous CIK cell infusions once every week via the common hepatic artery or peripheral veins. At least four infusions were given with >1 × 10^10^ CIK cells each. Further AFP levels were examined one and four weeks after TACE/RFA and—only in the study group—before each CIK cell transfer and once every four weeks within one year after CIK cell therapy.

Comparing AFP levels during followup to baseline levels before TACE/RFA, no significant decrease in AFP concentration could be observed in the control group. By contrast, AFP concentrations in patients additionally treated with CIK cells gradually decreased during followup. The differences between the two patient groups were significant (*P* < 0.05). Within the control group, nine patients experienced recurrence within one year; six of them had AFP concentrations slightly higher than the baseline level. In the study group three patients developed recurrence within one year, with two of them having AFP levels slightly higher than the baseline level.

In summary, the one-year recurrence rate was 7.14% in the study group, which was significantly lower than in the control group (23.1%; *P* = 0.04). Therefore, CIK therapy was able to reduce short-term tumor recurrence. Serum AFP levels were also reduced in patients after CIK cell transfusions indicating that a sustainable decrease of AFP might be useful to predict the clinical efficacy of CIK cell therapy.

Regarding these promising results, the researchers recently published another retrospective study including 174 HCC patients from January 1999 to April 2012 [42]. Eighty-nine patients in the TACE + RFA group were treated by TACE/RFA alone and 85 patients in the TACE + RFA + CIK group received additional CIK cell therapy. After sequential therapy with TACE and RFA, blood was collected from patients in the CIK group. CIK cells were prepared and reinfused i.v. two weeks later; four to 25 (median = 9) successive CIK cell infusions were given per patient.

No major complications occurred during treatment and the overall frequency of adverse effects was similar in both groups. The evaluation of the clinical efficacy was based on modified Response Evaluation Criteria in Solid Tumors (mRECIST) [43]. Differences in the short-term efficacy (three months after therapy) of the two treatment modalities were not significant. After a median followup of 78 months OS and PFS were calculated for both groups. The median survival time (MST) and the median PFS were significantly longer in the CIK group than in the control group (MST: 56 months versus 31 months, *P* = 0.023; PFS: 17 months versus 10 months, *P* < 0.001). The 3-, 5-, and 10-year OS was also significantly higher in the CIK group (*P* ≤ 0.005).

The results of this study suggest that additional CIK cell infusions can be beneficial for patients treated with sequential TACE/RFA. CIK cell therapy can prolong recurrence-free survival, which points to a safe and effective treatment option for patients with HCC.

Hao et al. published a nonrandomized controlled clinical trial combining only TACE with CIK cell therapy [44]. From 2005 to 2008, 146 patients with unresectable HCC were assigned by request either to the TACE group or to the combination group. No significant differences were found in the baseline characteristics between the two groups. At first, all patients were treated by TACE. The 72 patients in the combination group received four additional CIK cell transfusions as one course of CIK treatment with maximally four courses in one patient every year.

According to RECIST criteria, short-term responses were similar in both groups: 18% (13 patients) in the combination group and 14.9% (11 patients) in the TACE group had PR; 81.9% (59 patients) in the combination group and 85.1% (63 patients) in the TACE group had SD. The PFS in the combination group was significantly improved compared to the TACE group; the 6-month, 1-year, and 2-year PFS rates were 72.2%, 40.4%, and 25.3% in the combination group versus 34.8%, 7.7%, and 2.6% in the TACE group (*P* < 0.001). Among other factors, CIK cell therapy and times of TACE before disease progression were associated with PFS and OS as identified by univariate analysis. The median OS was significantly longer in the combination group (31 months) than in the TACE group (10 months; *P* < 0.001).

In this study, CIK immunotherapy was able to prolong PFS and OS in patients with unresectable HCC. Especially the times of TACE combined with CIK cell therapy were critical for the clinical outcome. This might be due to the low residual tumor burden after several courses of TACE on which CIK cells might be most effective.

In a recently published trial, i.p. perfusions of autologous CIK cells were combined with local radio frequency (RF) hyperthermia in 31 patients with advanced HCC [45]. Twice a week all patients received i.p. perfusions of 3.2 × 10^9^ to 3.6 × 10^9^ CIK cells with local RF hyperthermia performed two hours later. This treatment was repeated four times as one course. After one month a new treatment course was performed.

No serious adverse events were observed and mild adverse events resolved without intervention. Levels of peripheral blood lymphocytes, the AFP concentration, and the abdominal circumference were recorded every two weeks. The level of peripheral blood CD4^+^, CD3^+^CD8^+^, and CD3^+^CD56^+^ cells increased significantly after treatment with CIK cells (*P* < 0.05). The AFP level (*P* = 0.001) and the abdominal circumference (*P* = 0.002) significantly decreased. After a median followup of 8.3 months (range 2–12 months), the median TTP was 6.1 months and the median OS 8.5 months. The 3-, 6-, 9-months, and 1-year survival rates were 93.5%, 77.4%, 41.9%, and 17.4%, respectively.

Although a control group is missing in order to draw definite conclusions, the authors suggest a benefit of this treatment modality for patients with advanced HCC. They also emphasize the necessity of more clinical trials including higher numbers of patients to provide evidence and evaluate combinations with other therapeutic approaches.

A relatively large randomized controlled study on postoperative CIK immunotherapy was conducted from January 2000 to 2002 [46]. The aim of this study was to evaluate the clinical outcome of two different schedules of adjuvant CIK cell therapy. For this purpose, 127 patients with HCC, who underwent radical hepatic resection, were randomly assigned to three groups. The first group (CIK-I) comprised 41 patients who received three courses of CIK cell transfer, while the 43 patients in group CIK-II were given six courses of CIK cell transfer. The control group (*n* = 43) did not receive any postoperative adjuvant therapy. The patients were followedup for a relatively long period of five to seven years.

In total, five patients had to stop CIK therapy due to side effects. Still, no long-dated side effects could be observed. The DFS rates were significantly longer in the CIK-treated groups than in the control group (*P* < 0.005), but there were no significant differences between the CIK-I and the CIK-II groups (*P* = 0.345). Comparing the 5-year DFS rates, the results of the CIK-I and the CIK-II groups are relatively close together (23.3% and 19.4%), while the result of the control group is about half the percentage (11.2%). Interestingly, there were no significant differences in the OS rates (*P* = 0.884). Apart from the treatment modality, liver cirrhosis, tumor size, tumor differentiation, and vascular invasion were identified as significant factors influencing the DFS (*P* < 0.05).

This randomized study was conducted with a relatively high number of patients and a long followup. The authors demonstrate that adoptive CIK cell therapy can prevent or at least delay recurrence of HCC after hepatic resection. However, adjuvant CIK cell therapy does not seem to be able to improve the OS.

Similar to the study about *α*-Gal-pulsed DCs and CIK cells for the treatment of pancreatic cancer, the same authors tested this strategy on HCC [47]. Eighteen patients with clinical stage III HCC were included in this study and treated with conventional therapies. Nine patients were enrolled in the study group and nine patients in the control group.

Autologous tumor cells were synthesized with *α*-Gal epitopes, then bound by natural anti-Gal antibody, and finally lysed. DCs were induced from PBMCs and co-cultured with this *α*-Gal epitope-expressing HCC tumor cell lysate. CIK cells were co-cultured with these DCs.

CIK cell transfusions in the study group started three days after completion of the chemo- or radiotherapy and were then given every week for two to seven times.

CIK application was safe as no serious side effects were observed. Tumor-specific CIK cell therapy significantly prolonged survival—the median survival of the study group was 17.1 months versus 10.1 in the control group (*P* = 0.00121). Increases in CD3^+^, CD4^+^, CD45RO^+^, CD8^+^, and CD56^+^ cells could be detected in the patients' peripheral blood after CIK therapy.

In summary, this innovative strategy has great potential for specific and individual tumor therapy. It would yet be interesting to compare the clinical effect of these tumor-specific CIK cells to standard CIK cells in order to elucidate whether the coincubation with the *α*-Gal-pulsed DCs gives a significant benefit.

The clinical trials on CIK cells in HCC therapy are summarized in Table 2.

## 5. Colorectal Cancer

Colorectal cancer belongs to the most common and deadliest types of cancer [1]. On the one hand, the incidence of colorectal cancer is decreasing in some countries, for example, in the United States. This decrease is due to an increase in detection and removal of precancerous lesions. On the other hand, the incidence is increasing in some other countries, for example, in several Asian and Eastern European countries, probably due to several lifestyle factors such as increasing obesity and smoking. In general, nonlifestyle risk factors for colorectal cancer include age, genetic predisposition, and chronic inflammatory bowel disease.

To our knowledge the only clinical trial with CIK cells including colorectal cancer patients was published by Schmidt-Wolf et al. in 1999 [48]. Here, CIK cells were transfected with a plasmid containing the IL-2 gene. Ten patients with metastatic disease were included in this study—seven patients with colorectal cancer, two patients with lymphoma, and one patient with renal carcinoma. The patients received one to five i.v. infusions of IL-2-transfected CIK cells and five infusions of untransfected CIK cells; the second treatment cycle followed three weeks after the first one.

CIK cell therapy was well tolerated; only three patients developed WHO grade 2 fever. Transfected cells could be detected in the patients' blood for up to two weeks after immunotherapy. Moreover, significantly increased serum levels of IFN-*γ*, GM-CSF, and transforming growth factor-*β* (TGF-*β*) were measured (*P* < 0.05). Also an increase in the cytotoxicity of PBLs was observed following CIK cell therapy.

The clinical evaluation in this study was based on comparison of CT scans before and after treatment and on the WHO Handbook for reporting results of cancer treatment [49]. At the beginning of this study, all ten patients were in PD. After treatment six patients remained in PD, three patients had SD, and one patient had a complete response (CR) with no signs of tumor for at least four weeks. In conclusion, this clinical trial proved CIK cell administration to be safe and promising for patients with colorectal cancer.

## 6. Conclusions and Future Prospects

The twenty clinical studies discussed here prove CIK cell therapy to be an effective treatment option—after or along with conventional therapies—for patients with gastrointestinal tumors. In addition, as it was shown to be safe and not to induce major adverse effects, CIK cell therapy is a valuable alternative for patients not able or willing to tolerate side effects of conventional chemotherapy [24, 33]. Within the different studies, CIK cells were applied either via peripheral veins, i.p., or via the common hepatic artery and all approaches were well tolerated. Most probably, the best application depends on the tumor side itself and on the prevailing circumstances; for example, in case a catheter is introduced for TACE, the application of CIK cells through the same catheter is apparent. Still, as CIK cells are easily applicable i.v., this therapeutic approach is theoretically useful for most types of tumors.

Several studies showed that after CIK cell application the levels of CD3^+^, CD4^+^, CD3^+^CD8^+^, CD3^+^CD45RO^+^, and CD3^+^CD56^+^ cell populations and the CD4^+^/CD8^+^ ratio in the patients' blood increased, which indicates a boosting cellular immunity induced by CIK cell transfusions [22, 31, 35, 45]. The secretion of IFN-*γ* by PBMCs and the IFN-*γ* serum level, respectively, were also shown to increase after CIK cell therapy [31]. In the study of Qui and colleagues the increased INF-*γ* secretion lasted for the followup period of two years and elevated CD3^+^, CD4^+^, CD3^+^CD8^+^, CD3^+^CD45RO^+^, and CD3^+^CD56^+^ levels returned to normal six to nine months after CIK cell therapy [31]. Shi and colleagues detected significantly increased levels of CD3^+^CD8^+^, CD25^+^ and CD3^+^CD56^+^ cells during the whole followup period of up to 108 days [35]. Remarkably, in the study of Weng and colleagues the percentages of CD4^+^, CD3^+^, CD56^+^, and CD3^+^CD56^+^ cells were lower in recurrent patients [40]. Therefore, the immunomonitoring of these T-cell subtypes seems to be critical to predict the clinical response to CIK-cell therapy as increases in these T-cell subtypes suggest a prolonged TTP.

Some types of cancer such as HCC are closely associated with underlying viral infections (HBV, HCV) making the viral load an important prognosis factor. Therefore, one therapeutic goal is to decrease the viral load in order to improve the clinical outcome. In some clinical studies on CIK cell therapy for HCC, the viral load of HBV has been determined before and after CIK transfusions in order to evaluate the effect of CIK cells on viral replication. After CIK cell therapy, the HBV copies of DNA/mL decreased. This indicates a positive effect of CIK cells on viral infections and thus a clear benefit for patients with tumors and chronic viral disease [35, 39].

In summary, CIK cell therapy provides a promising approach in cancer therapy. CIK cells have a favorable biology with non-MHC-restricted tumor targeting and uncomplicated isolation and cultivation. In all studies, CIK cell therapy was well tolerated and superior to conventional therapies alone. There are several questions yet to be elucidated, for example, the optimal application schedule and the best therapeutic combination with conventional treatment modalities.

A few of the abovementioned studies are retrospective studies and many authors stated that more large-scale randomized controlled studies are needed. Indeed, these retrospective studies only give an idea of the effectivity of CIK cell therapy while the improved data quality of prospective clinical studies results in a higher significance. The great advantage of CIK cell therapy is that it is a personalized therapeutic approach—unfortunately, this makes CIK cell therapy very cost- and time-intensive, hampering the conduction of large prospective randomized trials.

There are many variations in the methodology and in the clinical evaluation between the research groups, which impede the comparison of the trials. The *ex vivo* protocols for the generation of CIK cells are heterogenous. For example, Jiang et al. generate CIK cells by addition of IFN-*γ* and IL-2 on day 0, anti-CD3 antibody and IL-1*α* on day 1, and then IL-2 every third day [21]. In contrast, Li et al. add anti-CD3 antibody, IL-1*α*, and IFN-*γ* at day 0, IL-2 after 24 hours, and then IFN-*γ* and IL-2 every five days [33]. Shi and colleagues do not use any IL-1 at all but add only IFN-*γ* at day 0 and anti-CD3 antibody and IL-2 at day 1 [35]. The same applies for the concentration of cytokines and the time of incubation till harvesting. Often not even information about the resulting phenotype composition in the CIK cell cultures is given, which makes the drawing of definite conclusions even more difficult. The International Registry on CIK Cells (IRCC) was established to collect data about CIK cell therapy and to set new standards on the report of results from CIK cell application [50]. Making the generation of CIK cells, the treatment, and the reporting of results more uniform will definitely push the application of CIK cell therapy forward and result in advantageous treatment options for cancer patients.

## Figures and Tables

**Table 1:** Clinical studies applying CIK cells for the treatment of gastric cancer.

Study	Number of patients	Therapy	Results	Conclusions
Jiang et al., 2006 [21]	57	Gastrectomy and chemotherapy; immunotherapy group: additional CIK infusions	Better survival rate of patients in immunotherapy group only in beginning—after 2 yrs no difference in survival between the groups	Combination of chemo- and CIK cell therapy superior to chemotherapy alone in patients with advanced gastric cancer

Shi et al., 2012 [22]	151	Gastrectomy and chemotherapy; immunotherapy group: additional CIK cell therapy	The mean CD3^+^, CD4^+^ level and the CD4^+^/CD8^+^ ratio increased in patients' blood up to 2 months after immunotherapy; no severe side effects; OS and DFS significantly better in immunotherapy group	CIK cell therapy can prolong DFS and OS; patients with intestinal-type tumors benefit most from CIK cell therapy as determined by retrospective subgroup analysis

Wang et al., 2013 [23]	42	Chemotherapy; immunotherapy group: additional i.p. perfusion of CIK cells	Reduced volume of 2-cycle peritoneal fluid drainage in immunotherapy group; no serious adverse reactions; prolonged TTP and higher OS in immunotherapy group	Superior efficacy of combination of chemo- and CIK cell therapy for advanced gastric cancer patients with ascites; CIK cell therapy can enhance immunological function and prolong survival

Jiang et al., 2008 [24]	60	Chemotherapy; immunotherapy group: additional CIK cell transfusions	The remission rate was 58.6% in the immunotherapy group and 45.2% in the control group; side effects of CIK cell transfusions were chills, fever, general malaise, nausea, and vomiting	CIK cell therapy can reduce clinical signs of elderly advanced gastric cancer patients; side effects can be treated with conventional therapy

Jiang et al., 2010 [25]	156	Chemotherapy; immunotherapy group: additional CIK cell therapy	Significantly longer 2- and 5-yr survival time	Better survival after CIK cell therapy; increasing the frequency of CIK cell therapy seems to be beneficial for survival

Zhao et al., 2013 [26]	165	Gastrectomy and chemotherapy; immunotherapy group: additional CIK cell therapy	Improved 5-yr PFS and OS in immunotherapy group; within the immunotherapy group, the frequency of CIK cell therapy, clinical stage, and follow-up therapy were the most important factors for PFS and OS	CIK cell therapy can prevent recurrence and improve survival in combination with gastrectomy and chemotherapy

CIK: cytokine-induced killer; OS: overall survival; DFS: disease-free survival; i.p.: intraperitoneal; TTP: time to progression; PFS: progression-free survival.

**Table 2:** Clinical studies applying CIK cells for the treatment of HCC.

Study	Number of patients	Therapy	Results	Conclusions
Takayama et al., 2000 [34]	150	Resection; Immunotherapy group: additional infusions of lymphocytes activated *in vitro* with rIL-2, and anti-CD3	Recurrence: 59% in immunotherapy group versus 77% in control group; TTP: 2.8 yrs in immunotherapy group versus 1.6 yrs in control group	Immunotherapy lowered risk of recurrence by 41%; the difference in OS was not significant; safe and feasible treatment

Shi et al., 2004 [35]	13	i.v. CIK transfusions	Increased proportions of CD3^+^CD8^+^, CD25^+^, and CD3^+^CD56^+^ cells in peripheral blood up to 108 d after immunotherapy; median HBV viral load decreased from 1.85 × 10^6^ to 1.41 × 10^5^ copies of DNA/mL in 3 months	CIK cells can efficiently improve the immunological status of HCC patients; CIK cells played important role in antiviral and antitumoral treatment

Zhang et al., 2005 [38]	17	Resection; CIK cell transfusion	Only one case described: decreased ascites; improvement of nausea, and vomiting; large lymphocyte infiltration in tumor	Significant enhancement of antitumor immunity; perform CIK therapy to eradicate remaining tumor cells after operation

Zhao et al., 2006 [39]	64	TACE/RFA; immunotherapy group: additional CIK infusions i.v. or via hepatic artery	After 1 yr followup: 29 of 33 patients in immunotherapy group and 23 of 31 patients in control group were recurrence-free; in 29 patients in the immunotherapy group and in only 1 patient in the control group the HBV DNA content was <1 × 10^3^	CIK therapy can prolong the recurrence-free time and fight HBV; CIK therapy after TACE/RFA is an effective therapeutic strategy for HCC

Weng et al., 2008 [40]	85	TACE/RFA; immunotherapy group: additional CIK infusions via hepatic artery	Increased proportions of CD3^+^, CD4^+^, CD56^+^, and CD3^+^CD56^+^ cells and the CD4^+^/CD8^+^ ratio—percentages were lower in recurrent patients than in nonrecurrent patients; recurrence: 31.1% in immunotherapy group versus 85.0% in control group	CIK cell therapy can reduce recurrence and improve survival rates; CIK transfusions can boost immunity of HCC patients

Pan et al., 2010 [41]	83	TACE/RFA; immunotherapy group: additional CIK cell transfusions i.v. or via common hepatic artery	Downtrend of AFP only in immunotherapy group; 1-yr recurrence rate 7.14% in immunotherapy group versus 23.1% in control group; percentage of patients with HBV DNA content <1 × 10^3^ copies/mL was 73.5% in the immunotherapy group versus 9.1% in the control group	CIK cell transfusions can decrease the 1-yr recurrence rate of HCC patients and reduce serum AFP levels, which may serve as a useful marker to predict clinical outcome after immunotherapy and TACE/RFA

Huang et al., 2013 [42]	174	TACE/RFA; immunotherapy group: additional i.v. CIK cell infusions	Significantly longer OS and PFS in immunotherapy group	Combination of TACE/RFA and CIK cell therapy is safe and can be an effective treatment modality

Hao et al., 2010 [44]	146	TACE; immunotherapy group: additional i.v. CIK cell transfusions	1-yr and 2-yr PFS rates: 40.4% and 25.3% in the immunotherapy group versus 7.7% and 2.6% in the control group; 1-yr and 2-yr OS rates: 71.9% and 62.4% in the immunotherapy group versus 42.8% and 18.8% in the control group; the times of TACE and CIK cell transfusions were independent prognostic factors for PFS and OS	Adjuvant CIK cell therapy can greatly improve the efficacy of TACE and prolong PFS and OS in HCC patients

Wang et al., 2013 [45]	31	RF hyperthermia; i.p. CIK cell perfusions	Significant increases in levels of CD4^+^, CD3^+^CD8^+^, and CD3^+^CD56^+^ cells in peripheral blood; AFP and abdominal circumference decreased; median TTP: 6.1 mo; 1-yr survival rate: 17.4%; median OS: 8.5 months	I.p. perfusions of CIK cells combined with local RF hyperthermia are safe, can improve immunology, and prolong survival of HCC patients

Hui et al., 2009 [46]	127	Resection; immunotherapy group I: additional 3 courses of CIK therapy; immunotherapy group II: additional 6 courses of CIK therapy	DFS rates significantly higher in CIK-treated groups than in control group; no statistical significance between immunotherapy group I and group II; no statistical significance in OS between the 3 groups	Postoperative CIK cell therapy can prolong DFS but not the OS rates; valuable therapeutic strategy for HCC patients to prevent recurrence

Qiu et al., 2011 [47]	18	Resection, radio-, chemo-, and interventional therapies; immunotherapy group: additional transfusion of CIK cells previously cocultured with *α*-Gal epitope-pulsed DCs	Survival was significantly prolonged: 17.1 months in the immunotherapy group versus 10.1 months in the control group; all patients in the immunotherapy group had systemic cytotoxicity in response to tumor lysate, decreased serum AFP, and increased levels of CD8^+^, CD45RO^+^, and CD56^+^ cells in peripheral blood	CIK therapy was safe and effective; new therapeutic approach has great potential in tumor therapy

CIK: cytokine-induced killer; HCC: hepatocellular carcinoma; rIL-2: recombinant Interleukin-2; anti-CD3: anti-CD3 antibody; TTP: time to progression; OS: overall survival; i.v.: intravenous; HBV: hepatitis B virus; TACE: transarterial chemoembolization; RFA: radiofrequency ablation; AFP: alpha fetoprotein; PFS: progression-free survival; i.p.: intraperitoneal; DFS: disease-free survival; *α*-Gal: *α*1,3-galactosyl; DC: dendritic cell.
